# Swimming motion of rod-shaped magnetotactic bacteria: the effects of shape and growing magnetic moment

**DOI:** 10.3389/fmicb.2014.00008

**Published:** 2014-01-30

**Authors:** Dali Kong, Wei Lin, Yongxin Pan, Keke Zhang

**Affiliations:** ^1^Department of Mathematical Sciences, University of ExeterExeter, UK; ^2^Biogeomagnetism Group, Paleomagnetism and Geochronology Laboratory, Key Laboratory of the Earth's Deep Interior, Institute of Geology and Institute of Geology and Geophysics, Chinese Academy of SciencesBeijing, China

**Keywords:** magnetotactic bacteria, rod-shape, stokes flow

## Abstract

We investigate the swimming motion of rod-shaped magnetotactic bacteria affiliated with the *Nitrospirae* phylum in a viscous liquid under the influence of an externally imposed, time-dependent magnetic field. By assuming that fluid motion driven by the translation and rotation of a swimming bacterium is of the Stokes type and that inertial effects of the motion are negligible, we derive a new system of the twelve coupled equations that govern both the motion and orientation of a swimming rod-shaped magnetotactic bacterium with a growing magnetic moment in the laboratory frame of reference. It is revealed that the initial pattern of swimming motion can be strongly affected by the rate of the growing magnetic moment. It is also revealed, through comparing mathematical solutions of the twelve coupled equations to the swimming motion observed in our laboratory experiments with rod-shaped magnetotactic bacteria, that the laboratory trajectories of the swimming motion can be approximately reproduced using an appropriate set of the parameters in our theoretical model.

## 1. Introduction

By converting the mechanical energy of convection-driven fluid motion into the ohmic dissipation taking place in the Earth's outer core, the geodynamo generates and sustains the geomagnetic field (Moffatt, 1978; Zhang and Schubert, 2000) that protects or affects a wide range of life, from human being to micro-scale organisms, on our planet Earth. The whole evolution has taken place in the presence of the Earth's magnetic field and therefore has brought about phenomena such as magnetotaxis and magnetoreception (Winklhofer, 2010) A particular class of living microorganisms is magnetotactic bacteria, first discovered nearly four decades ago by Blakemore (1975), which contain the magnetic crystal of a narrow size carrying permanent magnetization (Bazylinski and Frankel, 2004; Faivre and Schüler, 2008; Yan et al., 2012; Prozorov et al., 2013) that allow them to swim along the lines of the Earth's magnetic field. In other words, the majority of them are north-seeking in the northern hemisphere while south-seeking in the southern hemisphere (Blakemore et al., 1980; Kirschvink, 1980; Frankel, 1984).

It is now well known that, driven by rapid rotation of its helical flagellar filaments which generates torque, a magnetotactic bacterium swims in the form of helical fashion against the viscous drag and torque under the influence of an external magnetic field (Berg and Anderson, 1973; Jones and Aizawa, 1991). From a dynamical point of view, the swimming style and speed of magnetotactic bacteria would sensitively depend on its shape and the strength of its magnetization. The simplest model of swimming magnetotactic bacteria can be constructed upon making the following two assumptions: (1) the bacteria have perfect spherical geometry (Nogueira and Lins de Barros, 1995; Pan et al., 2009) and (2) their movement is extremely slow such that the Stokes approximation can be made (Batchelor, 1967; Koiller et al., 1996). A huge mathematical advantage of spherical geometry is that the Stokes solution is not only very simple but also available (Batchelor, 1967). The drag force **D**_μ_ on a translating spherical body is given by

(1) $$D_{\mu }=-6\pi \mu r_{0}v,$$

where *r*_0_ is the radius of the sphere, μ is the dynamical viscosity of the fluid and **v** is the translating velocity, while the viscous torque **T**_μ_ on a rotating spherical body is

(2) $$T_{\mu }=-8\pi \mu r_{0}^{3}\Omega ,$$

where **Ω** represents the angular velocity of its rotation. On the basis of these simple expressions for the viscous drag and torque of a spherical body, Nogueira and Lins de Barros (1995) derived a system of six simple ordinary differential equations that govern the motion of a swimming spherical magnetotactic bacterium. An important characteristic of the spherical model is, as clearly indicated by Equations (1, 2), that the size of its drag force **D**_μ_ and its viscous torque **T**_μ_ does not depend on the direction of its translation or rotation. Erglis et al. (2007) studied the swimming motion of the motile magnetotactic bacterium in a rotating magnetic field by assuming that the velocity of a bacterium is in the direction of its long axis.

While spherical geometry or the swimming direction in the direction of a symmetry axis would dramatically simplify the relevant mathematical analysis, it does not capture the key dynamics of swimming magnetotactic bacteria that are typically non-spherical and may swim in an arbitrary direction. By keeping inertial effects of the swimming motion and using a prolate spheroid with moderate eccentricity, Cui et al. (2012) derived a system of twelve coupled non-linear ordinary differential equations that govern both the motion and orientation of swimming non-spherical magnetotactic bacteria. It is noteworthy that, as a consequence of the strong stiffness associated with inertial effects, the twelve ordinary differential equations derived by Cui et al. (2012) are highly complicated and numerical integration of the system is quite slow.

**Figure 1:**
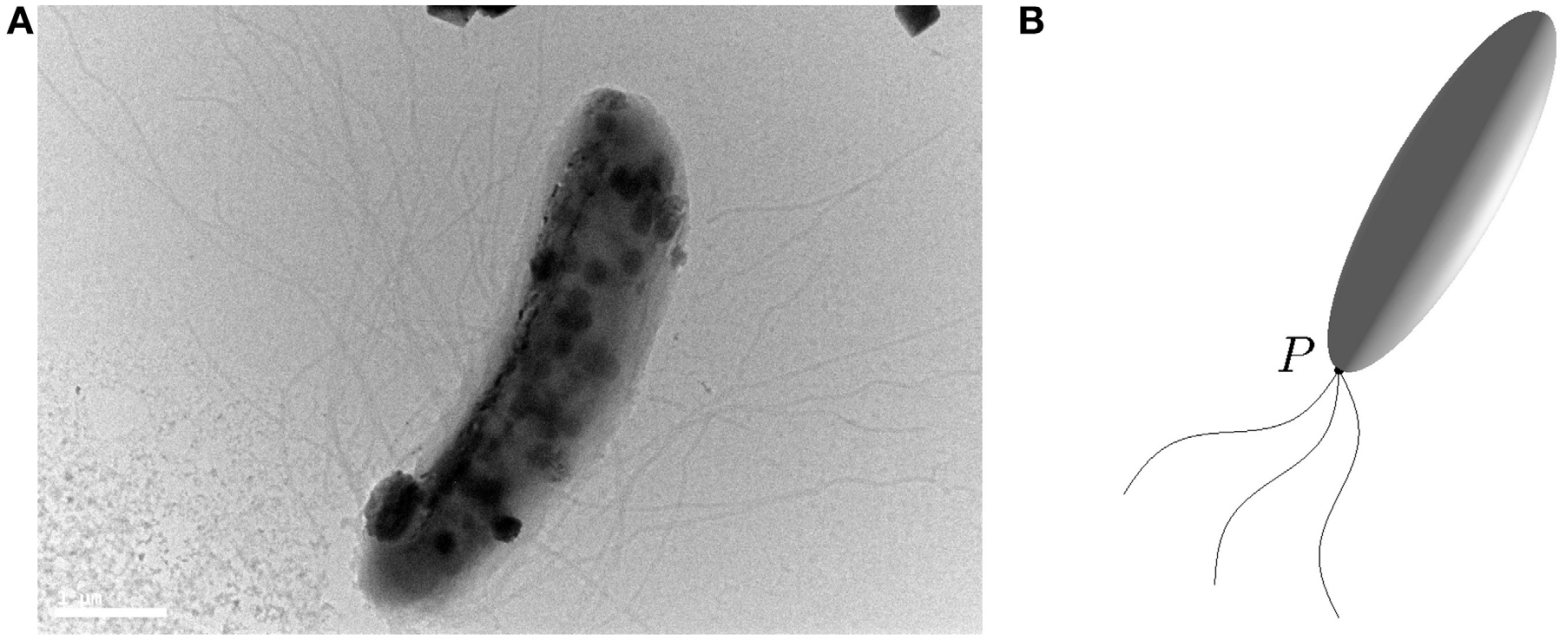
**(A)** A microscope image (Bar = 1μ m) of a rod-shaped magnetotactic bacterium found in Lake Miyun near Beijing, China. **(B)** An elongated prolate spheroid with eccentricity 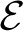 = 0.96 that provides an approximation of the rod-shaped bacterium for which its swimming motion is powered by the rapid rotation of helical flagellar filaments at a fixed point *P*.

Rod-shaped magnetotactic bacteria displayed in Figure 1A are found in Lake Miyun near Beijing, China. This type of magnetotactic bacteria, belonging to the *Nitrospirae* phylum, can synthesize hundreds of bullet-shaped magnetosomes in a single cell (Lin et al., 2011). It is recognized that the shape of this particular class of magnetotactic bacteria can be reasonably modeled by a strongly elongated prolate spheroid defined as

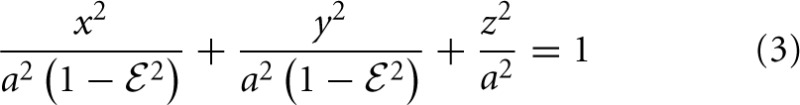



with its eccentricity 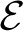 satisfying 0 < (1 − 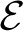) « 1, where *a* is the semi-major axis with 2*a* representing the length of a rod-shaped bacterium. As depicted in Figure 1B, the shape of the rod-shaped bacterium in Figure 1A can be approximately described by Equation (3) with 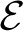 = 0.96.

The primary objective of this paper is to study, via both theoretical and experimental methods, the swimming motion of rod-shaped magnetotactic bacteria found in Lake Miyun (Lin et al., 2011). In comparison to the two previous studies—the spherical model by Nogueira and Lins de Barros (1995) and the inertial model by Cui et al. (2012)—there are three new elements in the present study. First, our non-spherical model accounts for, to leading-order approximation, the rod-shaped effect of swimming magnetotactic bacteria by taking the large eccentricity limit 0 < (1 − 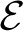) « 1 in Equation (3). As a consequence of rod-shaped geometry, the size of viscous torque, as we will show, is strongly dependent on the direction of rotation vector **Ω**. Second, by taking the time-dependent magnetic moment of magnetotactic bacteria, we attempt to model the dynamics of magnetotactic bacteria at the earlier stage of their growing phase when their magnetite formation is associated with a slow, diffusion-like process (Schüler and Baeuerlein, 1996). Third, we derive, by completely neglecting inertial effects, a new system of the twelve coupled equations that govern both the motion and orientation of rod-shaped magnetotactic bacteria in the laboratory frame of reference and that are much simpler than those of the inertial model derived by Cui et al. (2012).

It should be noticed that the analytical mathematical solutions for the motion of a viscous fluid due to a strongly prolate spheroid translating or rotating in an *arbitrary direction* are required to describe the swimming motion of rod-shaped magnetotactic bacteria. This is closely associated with a classical fluid dynamical problem which was first discussed by Jeffery (1922) and, then, comprehensively reviewed by Happel and Brenner (1965) in a research monograph. While the flow due to a strongly prolate spheroid translating or rotating in the direction *parallel to its symmetry axis* is relatively simply and has been used in various models (see, for example, Han et al., 2009), there exists no mathematical solutions that can be practically employed to study the dynamics of swimming motion of rod-shaped magnetotactic bacteria. This is because the existing solutions (Jeffery, 1922; Happel and Brenner, 1965) are based on the incomplete elliptic-type integrals that have to be evaluated numerically. We therefore need a set of the new analytical solutions that describe the fluid motion of a prolate spheroid translating or rotating in an arbitrary direction and that can be practically useful in modeling the swimming motion of rod-shaped magnetotactic bacteria.

In what follows we shall begin in section 2 by discussing the Stokes flow and the related viscous drag/torque for rod-shaped magnetotactic bacteria. This is followed by presenting our theoretical model and by deriving the twelve governing equations in section 3. Discussion of the results will be presented in section 4 and the paper closes in section 5 with a summary and some remarks.

## 2. Stokes flow, drag, and torque

### 2.1. Stokes flow for swimming rod-shaped bacteria

Stokes flow is referred to a class of fluid motion in that the speed of flow is extremely slow and the effect of viscosity is very strong such that inertial forces are much smaller comparing to viscous forces (Batchelor, 1967). In the language of fluid dynamics, the problem of swimming microorganisms is marked by a very small Reynolds number *Re* (Pureel, 1977), a dimensionless number defined as

$$Re=\frac{Ua\rho }{\mu },$$

where *U* is the typical velocity of the fluid motion, ρ is the liquid density, *a* denotes the typical length scale and μ is the dynamic viscosity of the liquid. Since the swimming speed *U* is very low and its characteristic dimension *a* is extremely small, the Stokes approximation, which neglects the inertial term in the Navier–Stokes equation by taking the limit *Re* → 0, is usually adopted for describing the motion of microorganisms (Koiller et al., 1996). It follows that the fluid motion generated by swimming rod-shaped magnetotactic bacteria is governed by the Stokes equation and the equation of continuity,

(4) $$\left\{ \begin{array}{l} \mu \nabla^{2}u=\nabla p,\\ \nabla \cdot u=0, \end{array}\right.$$

where **u** is the velocity of the flow and *p* is its pressure. For understanding the dynamics of swimming magnetotactic bacteria, it is necessary to have mathematical solutions of the Stokes flow created by both the translation and rotation of a rod-shaped body in an infinite expanse of viscous and incompressible fluid. In other words, we require the analytical solution to (Equation 4) subject to the condition that the fluid velocity **u** coincides with the bounding surface of a swimming magnetotactic bacterium at each of its points and **u** → 0 far away from the swimming bacterium.

It is important to notice that, while the mathematical problem of the spherical Stokes flow is classical, simple, two-dimensional and well-known (Batchelor, 1967), the Stokes flow associated with a rod-shaped swimming body is complicated, fully three-dimensional and not widely known. Various authors have considered the Stokes flow in non-spherical geometry. For example, Payne and Pell (1960) considered the Stokes problem in which the configuration of various obstacles has an axis of symmetry and the uniform flow at distant points is parallel to the symmetry axis. Kong et al. (2012) derived the first exact solution of Stokes flow for an arbitrarily rotating or translating oblate spheroid of arbitrary eccentricity using the Papkovich–Neuber formulation. In the following, we shall present the modified solution that is in a suitable form for our mathematical analysis of swimming rod-shaped bacteria in a viscous fluid.

### 2.2. Drag on translating rod-shaped bacteria at arbitrary angles

**Figure 2:**
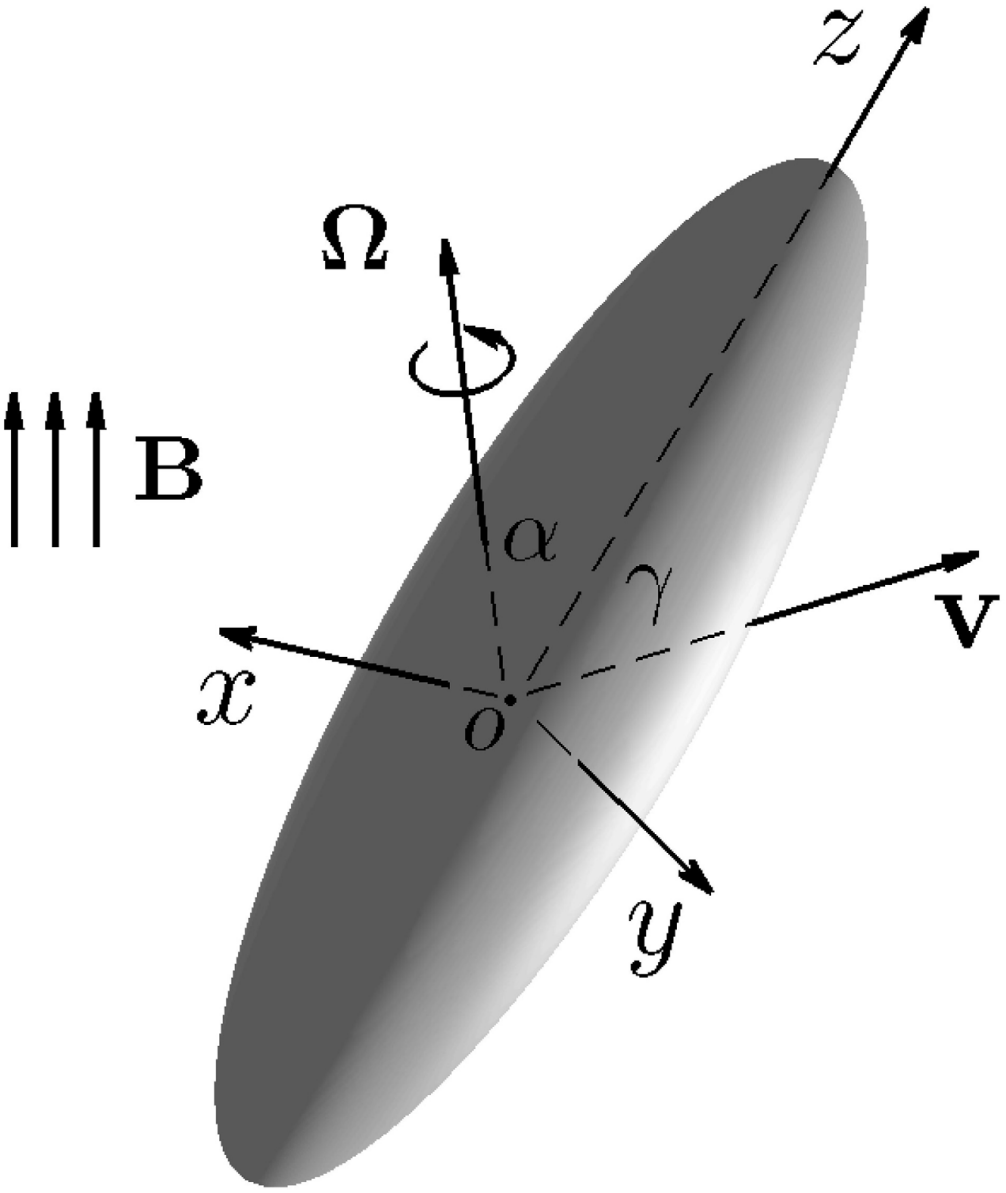
**Translation of a rod-shaped bacterium at an arbitrary angle of attack γ with the velocity **v**, where cartesian coordinates (*x, y, z*) are attached to the bacterium's body**. Rotation of a rod-shaped a rod-shaped bacterium with an arbitrary angle α and the angular velocity Ω under the influence of an externally imposed, time-dependent magnetic field **B**.

In order to describe the swimming motion of a rod-shaped bacterium, we require the mathematical solution of a three-dimensional Stokes flow driven by translating an elongated prolate spheroid with its eccentricity 0 < (1 − 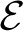) « 1 at an arbitrary angle of attack γ, the angle between the direction of the translating velocity **v** and the symmetry axis *z* of a rod-shaped bacterium, which is sketched in Figure 2. Note that cartesian coordinates (*x, y, z*) are attached to the bacterium's body. Our swimming model needs an analytical formula that expresses the viscous drag force **D**_*B*_ on the translating rod-shaped bacterium as a function of 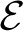 and γ.

Upon adopting the Papkovich–Neuber formulation (Papkovich, 1932; Neuber, 1934), the flow velocity **u** satisfying (Equation 4) can be written in the form

(5) $$\left\{ \begin{array}{l} u=\nabla (r\cdot \psi +\chi )-2\psi ,\\ p=2\mu (\nabla \cdot \psi ), \end{array}\right.$$

where **r** is the position vector, **ψ**, a vector harmonic function, satisfies ∇^2^**ψ** = 0 and χ, a scalar harmonic function, is a solution to ∇^2^χ = 0. Both **ψ** and χ can be obtained by using the expansion of prolate spheroidal harmonics (Kong et al., 2012). The main mathematical complication and difficulty in applying the Papkovich–Neuber formulation to the present problem stem from both non-spherical geometry/coordinates and three-dimensionality that make the analysis lengthy and cumbersome.

We first introduce oblate spheroidal coordinates defined by three sets of orthogonal level surfaces: the radial coordinate ξ ∈ [ξ_0_, ∞) characterizes oblate spheroidal surfaces

$$\frac{z^{2}}{c^{2}\xi^{2}}+\frac{x^{2}+y^{2}}{c^{2}\left(\xi^{2}-1\right)}=1,$$

the angular coordinate η ∈ [−1, 1] determines hyperboloids

$$\frac{z^{2}}{c^{2}\eta^{2}}-\frac{x^{2}+y^{2}}{c^{2}\left(1-\eta^{2}\right)}=1,$$

and, finally, the third coordinate is azimuthal angle ϕ which is the same as that in spherical polar coordinates. Here *c* is the common focal length for all the spheroids and hyperboloids, the bounding surface of an oblate spheroidal body (or a rod-shaped bacterium) is described by 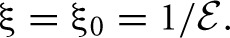


The domain of Stokes flow in the exterior of the prolate spheroid (or the rod-shaped bacterium) is then defined by {ξ_0_ ≤ ξ < ∞, −1 ≤ η ≤ 1, 0 ≤ φ ≤ 2π} while the transformation between prolate spheroidal coordinates (ξ, η, φ) and the corresponding cartesian coordinates (*x, y, z*) is given by

(6) $$\left\{ \begin{array}{l} x=c\sqrt{\left(\xi^{2}-1\right)\left(1-\eta^{2}\right)}\cos\varphi ,\\ y=c\sqrt{\left(\xi^{2}-1\right)\left(1-\eta^{2}\right)}\sin\varphi ,\\ z=c\xi \eta . \end{array}\right.$$

In this paper, we shall use ($\hat{\xi },\hat{\eta },\hat{\phi }$) to denote unit vectors in oblate spheroidal coordinates and ($\hat{x},\hat{y},\hat{z}$) as unit vectors in cartesian coordinates (*x, y, z*) depicted in Figure 2.

Suppose that a rod-shaped bacterium moves with the velocity **v** at the speed |**v**| written in the form

$$v=|v|\left[\left(\sin\gamma \cos\tilde{\phi }\right)\hat{x}+\left(\sin\gamma \sin\tilde{\phi }\right)\hat{y}+\left(\cos\gamma \right)\hat{z}\right],$$

where the angles γ and $\tilde{\phi }$ specify the direction of **v**. It can be shown, after some length mathematical analysis, that the three-dimensional solution **u**^*t*^ and the corresponding pressure *p*^*t*^ describing a Stokes flow driven by translating the bacterium with its velocity **v** at arbitrary angles γ and $\tilde{\phi }$ are

(7) $$\begin{array}{l} \frac{\hat{\xi }\cdot u^{t}}{\left|v\right|}=\sqrt{\frac{\xi^{2}-1}{\xi^{2}-\eta^{2}}}\;\eta \cos\gamma \\ \;\times \left[\frac{\hat{\xi }+\frac{2\xi }{\xi^{2}-1}}{\left(\xi_{0}^{2}+1\right){\hat{\xi }}_{0}-2\xi_{0}}+\frac{\hat{\xi }-\frac{2\xi }{\xi^{2}-1}}{\frac{\xi_{0}^{2}+1}{\xi_{0}^{2}}{\hat{\xi }}_{0}-\frac{2}{\xi_{0}}}\right]\\ \;+\frac{\sqrt{1-\eta^{2}}}{\sqrt{\xi^{2}-\eta^{2}}}\left[\frac{-\frac{\xi }{2}\hat{\xi }-1}{\frac{\xi_{0}^{2}-3}{4}{\hat{\xi }}_{0}-\frac{\xi_{0}}{2}}+\frac{\frac{\xi }{2}\hat{\xi }-\frac{\xi^{2}-2}{\xi^{2}-1}}{\frac{\xi_{0}^{2}-3}{2\left(\xi_{0}^{2}-1\right)}{\hat{\xi }}_{0}-\frac{\xi_{0}}{\xi_{0}^{2}-1}}\right]\\ \;\times \cos(\varphi -\tilde{\phi })\sin\gamma , \end{array}$$

(8) $$\begin{array}{l} \frac{\hat{\eta }\cdot u^{t}}{\left|v\right|}=\frac{\sqrt{1-\eta^{2}}}{\sqrt{\xi^{2}-\eta^{2}}}\cos\gamma \\ \;\times \left[\frac{\frac{\xi }{2}\hat{\xi }}{\frac{\xi_{0}^{2}+1}{2}{\hat{\xi }}_{0}-\xi_{0}}+\frac{\frac{\xi }{2}\hat{\xi }-1}{\frac{\xi_{0}^{2}+1}{2\xi_{0}^{2}}{\hat{\xi }}_{0}-\frac{1}{\xi_{0}}}\right]\\ \;+\frac{1}{\sqrt{\xi^{2}-\eta^{2}}}\left[\frac{\frac{\sqrt{\xi^{2}-1}}{2}\hat{\xi }}{\frac{\xi_{0}^{2}-3}{4}{\hat{\xi }}_{0}-\frac{\xi_{0}}{2}}-\frac{\frac{\sqrt{\xi^{2}-1}}{2}\hat{\xi }-\frac{\xi }{\sqrt{\xi^{2}-1}}}{\frac{\xi_{0}^{2}-3}{2\left(\xi_{0}^{2}-1\right)}{\hat{\xi }}_{0}-\frac{\xi_{0}}{\xi_{0}^{2}-1}}\right]\\ \;\times \eta \cos(\varphi -\tilde{\phi })\sin\gamma , \end{array}$$

(9) $$\begin{array}{l} \frac{\hat{\phi }\cdot u^{t}}{\left|v\right|}=\left[\frac{\frac{1}{2}\hat{\xi }}{\frac{\xi_{0}^{2}-3}{4}{\hat{\xi }}_{0}-\frac{\xi_{0}}{2}}-\frac{\frac{1}{2}\hat{\xi }-\frac{\xi }{\xi^{2}-1}}{\frac{\xi_{0}^{2}-3}{2\left(\xi_{0}^{2}-1\right)}{\hat{\xi }}_{0}-\frac{\xi_{0}}{\xi_{0}^{2}-1}}\right]\\ \;\times \sin(\varphi -\tilde{\phi })\sin\gamma , \end{array}$$

(10) $$\begin{array}{l} \frac{p^{t}}{\mu |v|}=-\frac{2}{c}[\frac{\xi }{\xi^{2}-\eta^{2}}\sqrt{\frac{1-\eta^{2}}{\xi^{2}-1}}\frac{\sin\gamma \cos(\varphi -\tilde{\phi })}{\frac{\xi_{0}^{2}-3}{4}{\hat{\xi }}_{0}-\frac{\xi_{0}}{2}}\\ \;+\frac{\eta }{\xi^{2}-\eta^{2}}\frac{\cos\gamma }{-\frac{\xi_{0}^{2}+1}{2}{\hat{\xi }}_{0}+\xi_{0}}], \end{array}$$

where

$${\hat{\xi }}_{0}=\ln\left[\frac{\left(\xi_{0}+1\right)}{\left(\xi_{0}-1\right)}\right];\;\hat{\xi }=\ln\left[\frac{\left(\xi +1\right)}{\left(\xi -1\right)}\right].$$

Here prolate spheroidal coordinates (ξ, η, φ) are employed for the convenience of computing the drag force that involves surface integration over the bounding surface of the bacterium. Analytical expressions (Equations 7–10) represent a solution satisfying both (Equation 4) and the non-slip boundary condition. It is evident that, apart from the special case with the attack angle γ = 0, this Stokes flow is fully three-dimensional.

With the availability of the three-dimensional solution (Equations 7–10), we are able to derive the drag force **D**_*B*_ on a swimming rod-shaped bacterium using cartesian coordinates (*x, y, z*) attached to the bacterium's body as sketched in Figure 2. The drag force **D**_*B*_ on a translating bacterium with the angle of attack γ can be expressed as

$$D_{B}=\int_{S}f^{t}\text{d}S,$$

where ∫_*S*_ denotes the surface integration over the bounding surface *S* of the bacterium, **f**^*t*^ in tensor notation is

(11) $$f_{i}^{t}=\left(-p^{t}\delta_{ij}+2\mu \sigma_{ij}^{t}\right)n_{j},$$

with δ_*ij*_ being the Dirac delta function, *n*_*j*_ being unit normal at the bounding surface *S* and

$$\sigma_{ij}^{t}=\frac{1}{2}\left(\frac{\partial u_{i}^{t}}{\partial x_{j}}+\frac{\partial u_{j}^{t}}{\partial x_{i}}\right).$$

**Figure 3:**
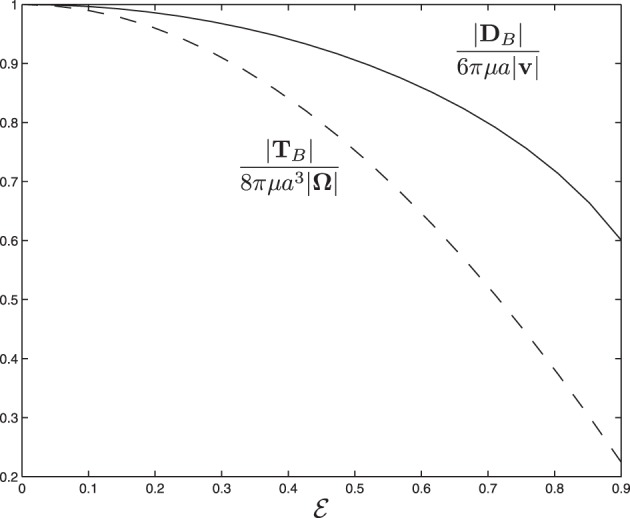
**The scaled drag force and the scaled viscous torque as a function of eccentricity** 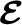 **for α = 45° and γ = 45°**.

The tensor σ*^t^_ij_* can be readily obtained from the expressions (7–9) by performing derivatives in prolate spheroidal coordinates. Evaluating *p*^*t*^ and σ*^t^_ij_* at the bounding surface ξ = ξ_0_ of the bacterium, integrating over its bounding surface *S*, we obtain an analytical formula for the drag force **D**_*B*_ on a translating bacterium:

(12) $$\begin{array}{l} \frac{D_{B}}{2\pi \mu c}=-[\frac{8+4\left(\xi_{0}^{2}-1\right)\left(-2+\xi_{0}{\hat{\xi }}_{0}\right)}{2\xi_{0}-\left(\xi_{0}^{2}-3\right){\hat{\xi }}_{0}}\\ \;+\frac{8\xi_{0}^{2}-4\xi_{0}\left(\xi_{0}^{2}-1\right){\hat{\xi }}_{0}}{2\xi_{0}-\left(\xi_{0}^{2}-3\right){\hat{\xi }}_{0}}]\left[(\hat{x}\cdot v)\hat{x}+(\hat{y}\cdot v)\hat{y}\right]\\ \;-[\frac{4\xi_{0}^{2}\left(\xi_{0}^{2}-1\right)\left(2-\frac{\xi_{0}^{2}-1}{\xi_{0}}{\hat{\xi }}_{0}\right)}{2\xi_{0}-2\xi_{0}^{3}+\left(\xi_{0}^{4}-1\right){\hat{\xi }}_{0}}\\ \;+\frac{4\left(\xi_{0}^{2}-1\right)\left(2-\xi_{0}{\hat{\xi }}_{0}\right)}{\xi_{0}-\left(\xi_{0}^{2}+1\right){\hat{\xi }}_{0}}](\hat{z}\cdot v)\hat{z}, \end{array}$$

which is valid for an arbitrary angle γ. Expression (12) will be used for constructing a set of the equations describing swimming rod-shaped bacteria. The dependence of the scaled drag force is tabulated in Table 1 as a function of γ for 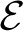 = 0.96 (or ξ_0_ = 1.0416667). Note that we have |**D**_*B*_|/(6πμ*a*|**v**|) → 1 in the spherical limit 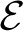 → 0 and that the size of the drag force is, as expected, significantly reduced as a result of the rod shaped body. Figure 3 shows how the drag force varies dramatically with the size of eccentricity 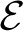 for γ = 45°, indicating that the dynamics of swimming rod-shaped bacteria would be quite different from that of spherical-shaped bacteria.

**Table 1:** **The scaled drag force and the scaled viscous torque as a function of γ and α for** 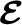 **= 0.96**.

**γ or α**	**|D_B_|/(6πμ*a*|*v*|)**	**|T_B_|/(8πμ*a*^3^|Ω|)**
0°	0.424447	0.057270
10°	0.428270	0.063091
20°	0.439094	0.077447
30°	0.455178	0.095336
40°	0.474163	0.113493
50°	0.493565	0.130060
60°	0.511126	0.143900
70°	0.525008	0.154267
80°	0.533874	0.160673
90°	0.536918	0.162839

*In the spherical limit* 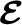 *→ 0 we obtain that |**D**_B_|/(6πμa|**v**|) → 1 and |**T**_B_|/(8πμa^3^|Ω|) → 1*.

### 2.3. Torque on a rotating rod-shaped bacteria at arbitrary angles

We also need the mathematical solution of a three-dimensional Stokes flow driven by rotating bacterium with the angular velocity **Ω** at an arbitrary angle α as sketched in Figure 2. Suppose that a bacterium is rotating with the angular velocity **Ω** in the form

$$\Omega =|\Omega |\left[\left(\sin\alpha \cos\beta \right)\hat{x}+\left(\sin\alpha \sin\beta \right)\hat{y}+\left(\cos\alpha \right)\hat{z}\right],$$

where the angles α and β specify the direction of **Ω** in the cartesian coordinates (*x, y, z*). A three-dimensional velocity **u**^*r*^ and the corresponding pressure *p*^*r*^ describing the Stokes flow driven by a rotating rod-shaped bacterium with the angular velocity **Ω** at arbitrary rotating angles α and β are

(13) $$\begin{array}{l} \frac{\hat{\xi }\cdot u^{r}}{c|\Omega |}=-\eta \sqrt{\frac{1-\eta^{2}}{\xi^{2}-\eta^{2}}}[\frac{\xi_{0}^{2}\hat{\xi }}{\left(\xi_{0}^{2}+1\right){\hat{\xi }}_{0}-2\xi_{0}}\\ \;-\frac{\frac{2\xi }{\xi^{2}-1}+\hat{\xi }}{\frac{\left(\xi_{0}^{2}+1\right)}{\xi_{0}^{2}-1}{\hat{\xi }}_{0}-\frac{2\xi_{0}}{\xi_{0}^{2}-1}}]\sin\alpha \sin(\varphi -\beta ), \end{array}$$

(14) $$\begin{array}{l} \frac{\hat{\eta }\cdot u^{r}}{c|\Omega |}=\frac{\sqrt{\xi^{2}-1}}{\sqrt{\xi^{2}-\eta^{2}}}[\frac{2\xi \hat{\xi }-4}{\frac{\left(\xi_{0}^{2}+1\right)}{\xi_{0}^{2}}{\hat{\xi }}_{0}-\frac{2}{\xi_{0}}}\\ \;-\frac{\xi \hat{\xi }-\frac{2\xi^{2}}{\xi^{2}-1}}{\frac{\left(\xi_{0}^{2}+1\right)}{\xi_{0}^{2}-1}{\hat{\xi }}_{0}-\frac{2\xi_{0}}{\xi_{0}^{2}-1}}]\sin\alpha \sin(\varphi -\beta ), \end{array}$$

(15) $$\begin{array}{l} \frac{\hat{\phi }\cdot u^{r}}{c|\Omega |}=\eta \left[\frac{\xi \hat{\xi }-\frac{2\xi^{2}}{\xi^{2}-1}}{\frac{\xi_{0}^{2}+1}{\xi_{0}^{2}-1}{\hat{\xi }}_{0}-\frac{2\xi_{0}}{\xi_{0}^{2}-1}}-\frac{2\xi \hat{\xi }-4}{\frac{\xi_{0}^{2}+1}{\xi_{0}^{2}}{\hat{\xi }}_{0}-\frac{2}{\xi_{0}}}\right]\\ \;\times \sin\alpha \cos(\varphi -\beta )\\ \;+\frac{\hat{\xi }-\frac{2\xi }{\xi^{2}-1}}{{\hat{\xi }}_{0}-\frac{2\xi_{0}}{\xi_{0}^{2}-1}}\sqrt{\left(\xi^{2}-1\right)\left(1-\eta^{2}\right)}\cos\alpha , \end{array}$$

(16) $$\frac{p^{r}}{\mu |\Omega |}=\frac{-8\eta }{\xi^{2}-\eta^{2}}\sqrt{\frac{1-\eta^{2}}{\xi^{2}-1}}\left[\frac{\sin\alpha \sin(\varphi -\beta )}{\left(\xi_{0}^{2}+1\right){\hat{\xi }}_{0}-2\xi_{0}}\right].$$

Evidently, apart from the special case with α = 0, the Stokes flow described by Equations (13–16) is fully three-dimensional.

The viscous torque **T**_*B*_ acting on the rotating bacterium can be expressible as

$$T_{B}=\int_{S}r_{S}\times f^{r}\text{d}S,$$

where **r**_*S*_ denotes the position vector for the bounding surface *S* of the bacterium and the viscous force **f**^*r*^ on the surface *S* is given by

(17) $$f_{i}^{r}=\left[-p^{r}\delta_{ij}+\mu \left(\frac{\partial u_{i}^{r}}{\partial x_{j}}+\frac{\partial u_{j}^{r}}{\partial x_{i}}\right)\right]n_{j},$$

with the velocity *u^r^_i_* and *p*^*r*^ given by Equations (13–16) evaluated at the outer surface ξ = ξ_0_ of the bacterium. After a lengthy analysis analogous to that for the drag **D**_*B*_, the torque **T**_*B*_ in cartesian coordinates (*x, y, z*) is found to be

(18) $$\begin{array}{l} \frac{T_{B}}{8\pi \mu c^{3}}=\frac{-1}{-2\xi_{0}+\left(\xi_{0}^{2}+1\right){\hat{\xi }}_{0}}\left[(\hat{x}\cdot \Omega )\hat{x}+(\hat{y}\cdot \Omega )\hat{y}\right]\\ \;\times [2\xi_{0}\left(\xi_{0}^{2}-1\right)\tanh^{-1}\frac{1}{\xi_{0}}\\ \;+\frac{-4+8\xi_{0}^{2}-3\xi_{0}\left(\xi_{0}^{2}-1\right){\hat{\xi }}_{0}}{3}]\\ \;+\frac{4}{3}\left[\frac{\left(\xi_{0}^{2}-1\right)}{-2\xi_{0}+\left(\xi_{0}^{2}-1\right){\hat{\xi }}_{0}}\right](\hat{z}\cdot \Omega )\hat{z}, \end{array}$$

which is valid for arbitrary rotating vector **Ω**. Here, for example,

$$\hat{x}\cdot \Omega =|\Omega |\left(\sin\alpha \cos\beta \right).$$

The dependence of the scaled torque is tabulated in Table 1 as a function of α for 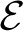 = 0.96. It can be seen that we have |**T**_*B*_|/(8πμ*a*^3^|Ω|) → 1 in the spherical limit 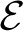 → 0 and that the size of the scaled torque is sensitively dependent on the rotating angle α, ranging from 0.057 at α = 0 to 0.163 at α = 90°. Figure 3 shows how the the viscous torque varies dramatically with the size of eccentricity 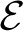 for α = 45°, indicating again that the dynamics of swimming rod-shaped bacteria would be quite different from that of spherical-shaped bacteria.

## 3. Model and governing equations

For deriving the equations governing the swimming motion of rod-shaped magnetotactic bacteria, we shall make the following six assumptions: (1) the geometry of a rod-shaped magnetotactic bacterium can be described by an elongated prolate spheroid (Equation 3) with 0 < (1 − 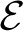) « 1; (2) the body of rod-shaped magnetotactic bacteria is non-deformable and, hence, the equation of rigid-body dynamics becomes applicable; (3) interaction between different magnetotactic bacteria during their swimming motion (Ishikawa et al., 2007), as clearly suggested by our laboratory experiments, is weak and, hence, can be negligible; (4) the translation and rotation of magnetotactic bacteria are powered by the rapid rotation of helical flagellar filaments at a fixed point *P*, as sketched in Figure 1B, which, as used and explained by Nogueira and Lins de Barros (1995), may be modeled a driving force **F**_*B*_ in the body frame:

(19) $$F_{B}=F_{12}\left[\cos(\omega_{0}t)\hat{x}+\sin(\omega_{0}t)\hat{y}\right]+F_{3}\hat{z},$$

where ω_0_ is the frequency of flagellum rotation while *F*_12_, *F*_3_, and ω_0_ may be regarded as parameters of the problem; (5) the inertial effects of swimming rod-shaped magnetotactic bacteria are small and, thus, negligible; and finally, (6) the initial phase of growing magnetic moment in a magnetotactic bacterium can be described by the equation

(20) $$m(t)=m_{0}\left[1-e^{-(t-t_{0})/\tau_{0}}\right],$$

where **m** is parallel to the symmetry axis of the magnetotactic bacterium, **m**_0_ denotes the magnetic moment in the limit *t* → ∞ and τ_0_ represents a parameter controlling the grow rate of the magnetic moment.

**Figure 4:**
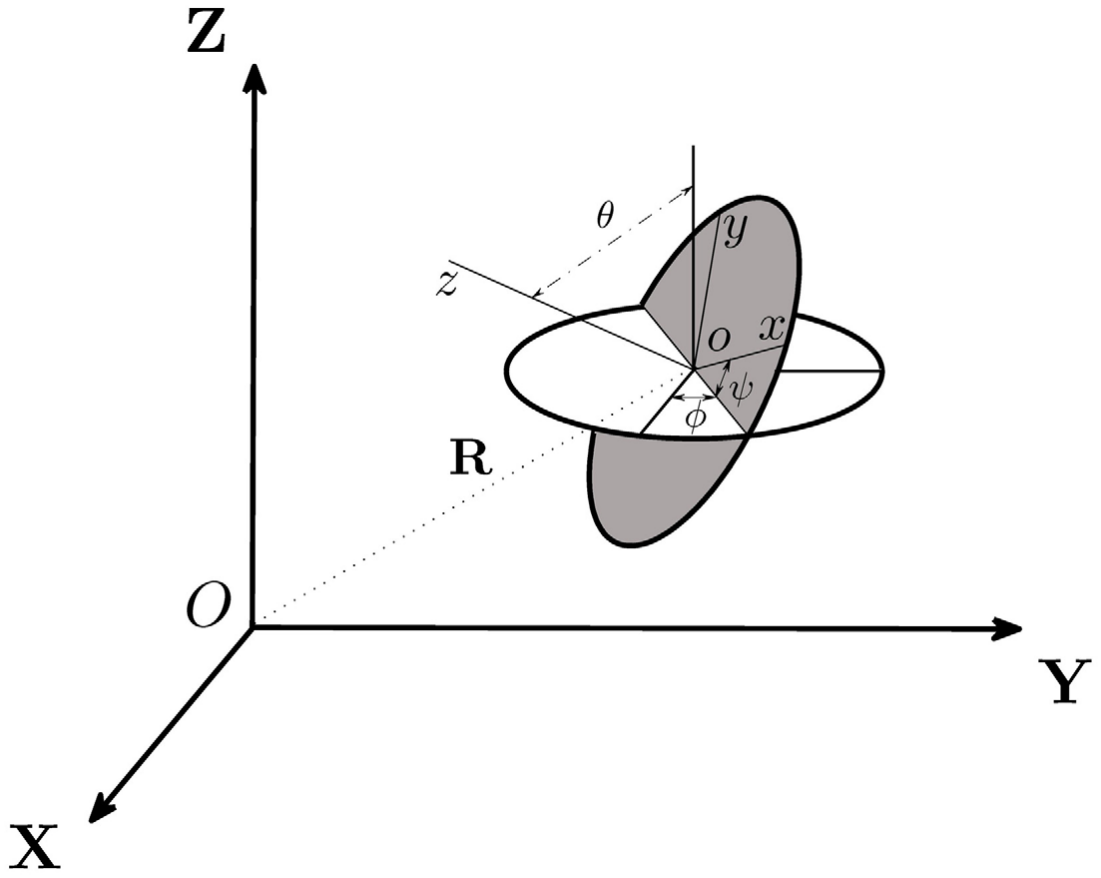
**Two cartesian coordinates, (*X, Y, Z*) and (*x, y, z*), used in our theoretical analysis, are related by the three Euler angles (θ, ψ, ϕ) and the position vector R**. Cartesian coordinates (*X, Y, Z*) represent a reference of frame fixed in the laboratory while (*x, y, z*) denote a reference of frame fixed in the body of the bacterium.

On the basis of the above six assumptions, we are able to derive the twelve coupled equations that govern the swimming motion of a rod-shaped magnetotactic bacterium with a time-dependent magnetic moment **m** in a viscous fluid. Two cartesian coordinates (*x, y, z*) and (*X, Y, Z*), which are sketched in Figure 4, are needed to describe the swimming motion. Cartesian coordinates (*x, y, z*) represent a reference of frame fixed in the bacterium's body with *z* at its symmetry axis (Figure 2); this reference will be referred to as the body frame. The position of the bacterium's center *o* in Figure 4 is described by the position vector

$$R=X\hat{X}+Y\hat{Y}+Z\hat{Z}$$

in cartesian coordinates (*X, Y, Z*) with the corresponding unit vectors ($\hat{X},\hat{Y},\hat{Z}$) fixed in a laboratory; this reference will be referred to as the laboratory frame.

A rod-shaped magnetotactic bacterium swims under the combined action of a propel force **F**_*B*_, a viscous drag **D**_*B*_, a viscous torque **T**_*B*_ and an externally imposed rotating magnetic field **B**. In this paper, subscript *B* denotes the quantity measured in the body frame of reference (*x, y, z*) while subscript *L* for the quantity described in the laboratory frame (*X, Y, Z*). The relative position between two cartesian coordinates, (*x, y, z*) and (*X, Y, Z*), is determined by six variables: the position vector **R** = (*X, Y, Z*) together with three Euler angles (θ, ϕ, ψ) illustrated in Figure 4. Additionally, we also introduce the three vectors: a translation vector **v**_*L*_ of a magnetotactic bacterium in the laboratory frame, a translation vector **v** of a magnetotactic bacterium in the body frame and a rotation vector Ω in the body frame. There exist 12 degrees of freedom that determine the swimming motion of a rod-shaped magnetotactic bacterium: (1) the three position coordinates **R** = (*X, Y, Z*) in the laboratory frame; (2) the three components of its velocity vector **v**_*L*_ = (**v**_*L*_ · $\hat{X}$, **v**_*L*_ · $\hat{Y}$, **v**_*L*_ · $\hat{Z}$) in the laboratory frame; (3) the three components of the angular velocity Ω = ($\hat{x}$ · Ω, $\hat{y}$ · Ω, $\hat{z}$ · Ω) in the body frame and, finally, (iv) the three Euler angles (θ, ϕ, ψ).

The first set of the governing equations is derived from Newton's second law stating that the rate of change of the momentum must be equal to the sum of all external forces acting on it,

$$M\frac{\text{d}v_{L}}{\text{d}t}=F_{L}+D_{L},$$

where **F**_*L*_ and **D**_*L*_ are the propel and viscous forces in the laboratory frame. Upon neglecting inertial effects because the mass *M* of a rod-shaped magnetotactic bacterium (which is about 9.5 × 10^−16^ Kg) is extremely small, we may rewrite the above equation as

(21) $$0=F_{B}+D_{B}.$$

Note that, by neglecting inertial effects, the forces in the body frame (**F**_*B*_, **D**_*B*_) are the same as those in the laboratory frame (**F**_*L*_, **D**_*L*_). Equation (21) can be solved exactly, after making use of Equations (12, 19), to give the three velocity components in the body frame of reference

(22) $$\begin{array}{l} v\cdot \hat{x}=\frac{F_{12}\cos\omega_{0}t}{2\pi \mu c}[\frac{8+4\left(\xi_{0}^{2}-1\right)\left(-2+\xi_{0}{\hat{\xi }}_{0}\right)}{2\xi_{0}-\left(\xi_{0}^{2}-3\right){\hat{\xi }}_{0}}\\ \;+{\frac{8\xi_{0}^{2}-4\xi_{0}\left(\xi_{0}^{2}-1\right){\hat{\xi }}_{0}}{2\xi_{0}-\left(\xi_{0}^{2}-3\right){\hat{\xi }}_{0}}]}^{-1}, \end{array}$$

(23) $$\begin{array}{l} v\cdot \hat{y}=\frac{F_{12}\sin\omega_{0}t}{2\pi \mu c}[\frac{8+4\left(\xi_{0}^{2}-1\right)\left(-2+\xi_{0}{\hat{\xi }}_{0}\right)}{2\xi_{0}-\left(\xi_{0}^{2}-3\right){\hat{\xi }}_{0}}\\ \;+{\frac{8\xi_{0}^{2}-4\xi_{0}\left(\xi_{0}^{2}-1\right){\hat{\xi }}_{0}}{2\xi_{0}-\left(\xi_{0}^{2}-3\right){\hat{\xi }}_{0}}]}^{-1}, \end{array}$$

(24) $$\begin{array}{l} v\cdot \hat{z}=\frac{F_{3}\sin\omega_{0}t}{2\pi \mu c}[\frac{4\xi_{0}^{2}\left(\xi_{0}^{2}-1\right)\left(2\xi_{0}-\left(\xi_{0}^{2}-1\right){\hat{\xi }}_{0}\right)}{2\xi_{0}^{2}\left(1-\xi_{0}^{2}\right)+\xi_{0}\left(\xi_{0}^{4}-1\right){\hat{\xi }}_{0}}\\ \;+{\frac{4\left(\xi_{0}^{2}-1\right)\left(2-\xi_{0}{\hat{\xi }}_{0}\right)}{\xi_{0}-\left(\xi_{0}^{2}+1\right){\hat{\xi }}_{0}}]}^{-1}. \end{array}$$

The velocity **v** in the body frame needs to be transformed to the laboratory frame of reference, denoted as **v**_*L*_ by using the three Euler angles, which is expressible as

(25) $$\begin{array}{l} v_{L}\cdot \hat{X}=v\cdot \hat{x}\left(\cos\phi \cos\psi -\sin\phi \cos\theta \sin\psi \right)\\ \;-v\cdot \hat{y}\left(\cos\phi \sin\psi +\sin\phi \cos\theta \cos\psi \right)\\ \;+v\cdot \hat{z}\left(\sin\phi \sin\theta \right), \end{array}$$

(26) $$\begin{array}{l} v_{L}\cdot \hat{Y}=v\cdot \hat{x}\left(\sin\phi \cos\psi +\cos\phi \cos\theta \sin\psi \right)\\ \;-v\cdot \hat{y}\left(\sin\phi \sin\psi -\cos\phi \cos\theta \cos\psi \right)\\ \;-v\cdot \hat{z}\left(\cos\phi \sin\theta \right), \end{array}$$

(27) $$\begin{array}{l} v_{L}\cdot \hat{Z}=v\cdot \hat{x}\left(\sin\theta \sin\psi \right)+v\cdot \hat{y}\left(\sin\theta \cos\psi \right)\\ \;+v\cdot \hat{z}\cos\theta . \end{array}$$

Note that the three Euler angles are a function of time: θ = θ(*t*), ψ = ψ(*t*), and ϕ = ϕ(*t*). The second set of three equations is derived by relating the position of the bacterium **R** in the laboratory frame to its translation velocity **v**_*L*_,

$$\left(\hat{X}\frac{\text{d}X}{\text{d}t}+\hat{Y}\frac{\text{d}Y}{\text{d}t}+\hat{Z}\frac{\text{d}Z}{\text{d}t}\right)=v_{L}.$$

After making use of Equations (22–24) and (25–27), we can derive the following three equations for determining the position vector **R** = (*X, Y, Z*) in the laboratory frame of reference:

(28) $$\begin{array}{l} \frac{\text{d}X}{\text{d}t}=\frac{F_{12}}{2\pi \mu c}[\frac{8+4\left(\xi_{0}^{2}-1\right)\left(-2+\xi_{0}{\hat{\xi }}_{0}\right)}{2\xi_{0}-\left(\xi_{0}^{2}-3\right){\hat{\xi }}_{0}}\\ \;+{\frac{8\xi_{0}^{2}-4\xi_{0}\left(\xi_{0}^{2}-1\right){\hat{\xi }}_{0}}{2\xi_{0}-\left(\xi_{0}^{2}-3\right){\hat{\xi }}_{0}}]}^{-1}\\ \;\times \left[\cos\phi \cos(\omega_{0}t+\psi )-\sin\phi \cos\theta \sin(\omega_{0}t+\psi )\right]\\ \;+\frac{F_{3}}{2\pi \mu c}[\frac{4\xi_{0}^{2}\left(\xi_{0}^{2}-1\right)\left(2\xi_{0}-\left(\xi_{0}^{2}-1\right){\hat{\xi }}_{0}\right)}{2\xi_{0}^{2}\left(1-\xi_{0}^{2}\right)+\xi_{0}\left(\xi_{0}^{4}-1\right){\hat{\xi }}_{0}}\\ \;+{\frac{4\left(\xi_{0}^{2}-1\right)\left(2-\xi_{0}{\hat{\xi }}_{0}\right)}{\xi_{0}-\left(\xi_{0}^{2}+1\right){\hat{\xi }}_{0}}\;]}^{-1}\sin\phi \sin\theta , \end{array}$$

(29) $$\begin{array}{l} \frac{\text{d}Y}{\text{d}t}=\frac{F_{12}}{2\pi \mu c}[\frac{8+4\left(\xi_{0}^{2}-1\right)\left(-2+\xi_{0}{\hat{\xi }}_{0}\right)}{2\xi_{0}-\left(\xi_{0}^{2}-3\right){\hat{\xi }}_{0}}\\ \;+{\frac{8\xi_{0}^{2}-4\xi_{0}\left(\xi_{0}^{2}-1\right){\hat{\xi }}_{0}}{2\xi_{0}-\left(\xi_{0}^{2}-3\right){\hat{\xi }}_{0}}]}^{-1}\\ \;\times \left[\sin\phi \cos(\omega_{0}t+\psi )+\cos\phi \cos\theta \sin(\omega_{0}t+\psi )\right]\\ \;-\frac{F_{3}}{2\pi \mu c}[\frac{4\xi_{0}^{2}\left(\xi_{0}^{2}-1\right)\left(2\xi_{0}-\left(\xi_{0}^{2}-1\right){\hat{\xi }}_{0}\right)}{2\xi_{0}^{2}\left(1-\xi_{0}^{2}\right)+\xi_{0}\left(\xi_{0}^{4}-1\right){\hat{\xi }}_{0}}\\ \;+{\frac{4\left(\xi_{0}^{2}-1\right)\left(2-\xi_{0}{\hat{\xi }}_{0}\right)}{\xi_{0}-\left(\xi_{0}^{2}+1\right){\hat{\xi }}_{0}}]}^{-1}\cos\phi \sin\theta , \end{array}$$

(30) $$\begin{array}{l} \frac{\text{d}Z}{\text{d}t}=\frac{F_{12}}{2\pi \mu c}[\frac{8+4\left(\xi_{0}^{2}-1\right)\left(-2+\xi_{0}{\hat{\xi }}_{0}\right)}{2\xi_{0}-\left(\xi_{0}^{2}-3\right){\hat{\xi }}_{0}}\\ \;+{\frac{8\xi_{0}^{2}-4\xi_{0}\left(\xi_{0}^{2}-1\right){\hat{\xi }}_{0}}{2\xi_{0}-\left(\xi_{0}^{2}-3\right){\hat{\xi }}_{0}}]}^{-1}\\ \;\times \sin\theta \sin(\omega_{0}t+\psi )\\ \;-\frac{F_{3}}{2\pi \mu c}[\frac{4\xi_{0}^{2}\left(\xi_{0}^{2}-1\right)\left(2\xi_{0}-\left(\xi_{0}^{2}-1\right){\hat{\xi }}_{0}\right)}{2\xi_{0}^{2}\left(1-\xi_{0}^{2}\right)+\xi_{0}\left(\xi_{0}^{4}-1\right){\hat{\xi }}_{0}}\\ \;+{\frac{4\left(\xi_{0}^{2}-1\right)\left(2-\xi_{0}{\hat{\xi }}_{0}\right)}{\xi_{0}-\left(\xi_{0}^{2}+1\right){\hat{\xi }}_{0}}]}^{-1}\cos\theta . \end{array}$$

The third set of the equations is derived from the rotational dynamics of the angular momentum **L** which is

$$L=I_{x}\Omega_{x}\hat{x}+I_{y}\Omega_{x}\hat{y}+I_{z}\Omega_{z}\hat{z},$$

where (*I_x_*, *I_y_*, *I_z_*) denote the three principle moments of inertia of a rod-shaped bacterium. It is known that the rate of change of **L** must be equal to the sum of all torques acting on the rod-shaped bacterium:

$$\begin{array}{l} \left(I_{x}\hat{x}\frac{\text{d}\Omega_{x}}{\text{d}t}+I_{y}\hat{y}\frac{\text{d}\Omega_{y}}{\text{d}t}+I_{z}\hat{z}\frac{\text{d}\Omega_{z}}{\text{d}t}\right)+\Omega \times L\\ \;=T_{F}+T_{c}+T_{B}+T_{M}, \end{array}$$

where **T**_*F*_ represents the torque imposed by the driving force **F**_*B*_,

$$T_{F}=-a\hat{z}\times F_{B}=aF_{12}\left(\hat{x}\sin\omega_{0}t-\hat{y}\cos\omega_{0}t\right),$$

**T**_*c*_ = −*N_c_*$\hat{z}$ is related to the reaction couple of the flagellar rotation (Nogueira and Lins de Barros, 1995), the viscous torque **T**_*B*_ in the body frame is given by Equation (18) and the time-dependent magnetic torque in the laboratory frame is

$$\begin{array}{l} {\left(T_{M}\right)}_{\text{lab}}=m(t)\times B=(m_{0}B_{0})\left[1-e^{-(t-t_{0})/\tau }\right]\\ \;\hat{z}\times \left(\hat{X}\cos\Omega_{0}t+\hat{Y}\cos\Omega_{0}t\right), \end{array}$$

where *B*_0_ is the amplitude of the externally imposed, rotating magnetic field **B** and Ω_0_ denotes the frequency of the magnetic field **B**. Since all the torques must be expressed in the same frame of reference, we need to transform (**T**_*M*_)_lab_ in the laboratory frame to that in the body frame of reference using the three Euler angles. In the body frame, the magnetic torque **T**_*M*_ is

(31) $$\begin{array}{l} T_{M}=(m_{0}B_{0})\left[1-e^{-(t-t_{0})/\tau }\right]\\ \;\{[\left(\sin\psi \cos\phi +\sin\phi \cos\theta \cos\psi \right)\cos\Omega_{0}t\\ \;+\left(\sin\psi \sin\phi -\cos\phi \cos\theta \cos\psi \right)\sin\Omega_{0}t]\hat{x}\\ \;+[\left(\cos\psi \cos\phi -\sin\phi \cos\theta \sin\psi \right)\cos\Omega_{0}t\\ \;+\left(\cos\psi \sin\phi +\cos\phi \cos\theta \sin\psi \right)\sin\Omega_{0}t]\hat{y}\}. \end{array}$$

Furthermore, because of the extremely small moments of inertia (*I_x_*, *I_y_*, *I_z_*) in association with the small mass of the bacterium, we shall neglect inertial effects by writing the angular momentum equation as

(32) $$0=T_{F}+T_{c}+T_{M}+T_{B}.$$

This approximation dramatically simplifies the analysis and allows us to solve the vector equation (32) for the three components of **Ω** in the body frame of reference, which are

(33) $$\begin{array}{l} \hat{x}\cdot \Omega =\frac{-2\xi_{0}+\left(\xi_{0}^{2}+1\right){\hat{\xi }}_{0}}{8\pi \mu c^{3}}\\ \;\{aF_{12}\sin\omega_{0}t+m_{0}B_{0}\left(1-e^{-(t-t_{0})/\tau }\right)\\ \;\times [\left(\sin\psi \cos\phi +\sin\phi \cos\theta \cos\psi \right)\cos\Omega_{0}t\\ \;+\left(\sin\psi \sin\phi -\cos\phi \cos\theta \cos\psi \right)\sin\Omega_{0}t]\}\\ \;\times [2\xi_{0}\left(\xi_{0}^{2}-1\right)\tanh^{-1}{\frac{1}{\xi }}_{0}\\ \;+{\frac{1}{3}\left(-4+8\xi_{0}^{2}-3\xi_{0}\left(\xi_{0}^{2}-1\right){\hat{\xi }}_{0}\right)]}^{-1}, \end{array}$$

(34) $$\begin{array}{l} \hat{y}\cdot \Omega =\frac{-2\xi_{0}+\left(\xi_{0}^{2}+1\right){\hat{\xi }}_{0}}{8\pi \mu c^{3}}\\ \;\{-aF_{12}\cos\omega_{0}t+m_{0}B_{0}\left(1-e^{-(t-t_{0})/\tau }\right)\\ \;\times [\left(\cos\psi \cos\phi -\sin\phi \cos\theta \sin\psi \right)\cos\Omega_{0}t\\ \;+\left(\cos\psi \sin\phi +\cos\phi \cos\theta \sin\psi \right)\sin\Omega_{0}t]\}\\ \;\times [2\xi_{0}\left(\xi_{0}^{2}-1\right)\tanh^{-1}{\frac{1}{\xi }}_{0}\\ \;+{\frac{1}{3}\left(-4+8\xi_{0}^{2}-3\xi_{0}\left(\xi_{0}^{2}-1\right){\hat{\xi }}_{0}\right)]}^{-1}, \end{array}$$

(35) $$\hat{z}\cdot \Omega =\frac{3N_{c}\left[-2\xi_{0}+\left(\xi_{0}^{2}-1\right){\hat{\xi }}_{0}\right]}{32\pi \mu c^{3}\left(\xi_{0}^{2}-1\right)}.$$

While (*X, Y, Z*) in connection with (Equations 28–30) leads to the position of the center *o* of a rod-shaped bacterium in the laboratory frame, its orientation, which is related to the angular velocity **Ω**, is described by the three Euler angles governed by the following three equations

(36) $$\frac{\text{d}\theta }{\text{d}t}=\hat{x}\cdot \Omega \cos(\psi )-\hat{y}\cdot \Omega \sin(\psi ),$$

(37) $$\frac{\text{d}\phi }{\text{d}t}=\hat{x}\cdot \Omega \csc(\theta )\sin(\psi )+\hat{y}\cdot \Omega \csc(\theta )\cos(\psi ),$$

(38) $$\begin{array}{l} \frac{\text{d}\psi }{\text{d}t}=-\hat{x}\cdot \Omega \cot(\theta )\sin(\psi )\\ \;-\hat{y}\cdot \Omega \cot(\theta )\cos(\psi )+\hat{z}\cdot \Omega , \end{array}$$

where $\hat{x}$ · **Ω**, $\hat{y}$ · **Ω**, and $\hat{z}$ · **Ω** are given by Equations (33–35).

The swimming motion of a rod-shaped magnetotactic bacterium at any instant *t*, starting from an initial condition at *t* = *t*_0_, can be modeled by mathematical solutions to the twelve coupled equations: (25–27) provide the three components of its velocity **v**_*L*_ in the laboratory frame; (Equations 28–30) lead to its position in the laboratory frame; (Equations 33–35) give its rotation vector **Ω**; and (Equations 36–38) describe its orientation in the laboratory frame. Although the twelve coupled equations are non-linear and coupled, they represent a mathematically tractable system enabling us to understand the motion of a swimming rod-shaped magnetotactic bacterium under the influence of an imposed magnetic field in laboratory experiments. In this study, we have solved the twelve coupled equations using Runge–Kutta–Fehlberg 4(5) method with an adaptive time step in which the accuracy is of the order *h*^4^ while an error estimator is of the order *h*^5^.

## 4. Results

The swimming motion of rod-shaped magnetotactic bacteria is investigated through both theoretical and experimental methods. Theoretically, for given a set of the model and physical parameters together with an appropriate initial condition, the twelve governing equations are numerically solved to determine the trajectories of swimming motion in the laboratory frame. Experimentally, we record the swimming motion of rod-shaped magnetotactic bacteria found in Lake Miyun, which is illustrated in Figure 1A, using charge-coupled device camera under an imposed, time-dependent magnetic field **B** in the laboratory frame (see Figure 2) given by

(39) $$B=B_{0}\left[\cos(\Omega_{0}t)\hat{X}+\sin(\Omega_{0}t)\hat{Y}\right],$$

where *B*_0_ and Ω_0_ are changeable in our laboratory experiments.

A set of the model parameters, geometric or physical, must be specified in order to solve the twelve coupled equations. Several parameters may be regarded as being well known but some are poorly determined. For example, the typical length 2*a* of rod-shaped magnetotactic bacteria and its magnetic moment *m*_0_ can be approximately measured or deduced in a reasonably accurate way. Other parameters such as the amplitude *F*_12_, *F*_3_, and the frequency ω_0_ have to be treated as the model parameters of a theoretical problem. The set of parameters used in calculating the swimming motion of rod-shaped magnetotactic bacteria found in Lake Miyun is listed in Table 2.

**Table 2:** **The values of physical/model parameters for the rod-shaped magnetotactic bacteria used in our calculation**.

**Parameter**	**Value**
The length of the bacterium	2*a* = 5 × 10^−6^ m
The mass of the bacterium	*M* = 9.5 × 10^−16^ Kg
Eccentricity	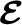 = 0.96
Focal length	*c* = *a* 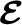 = 2.4 × 10^−6^
Outer surface coordinate	ξ_0_ = 1/ 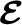 = 1.0417
Dynamical viscosity for water	μ = 10^−3^ Pa S
Magnetic moment	*m*_0_ = 10^−14^ A/m^2^
Frequency in Equation (19)	ω_0_ = 51/s
Force amplitude in Equation (19)	*F*_12_ = 1.1386 × 10^−12^ N
Force amplitude in Equation (19)	*F*_3_ = 8.8849 × 10^−12^ N
Reaction couple	*N*_*c*_ = 2.249 × 10^−19^ N m

An important quantity is the angle Φ_*B*_ (0 ≤ Φ_*B*_ ≤ 180°) between the symmetry axis *z* (or the direction of the magnetic moment **m** which is aligned with the symmetry axis *z*, see Figure 2) and the direction of the imposed magnetic field **B** during the swimming motion of a rod-shape magnetotactic bacterium. The size of the angle Φ_*B*_ cannot be estimated in our laboratory experiments but can be readily computed in theoretical experiments. In particular, we are interested in how the angle Φ_*B*_ varies during the growing phase of the bacterium's magnetic moment. Two different phases can be identified in our theoretical model. During the initial growing phase, marked by (*t* − *t*_0_)/τ_0_ < O(1) in Equation (20), when the magnetic moment |**m**| is weak, the angle Φ_*B*_ is hardly affected by the existence the magnetic moment and, hence, the angle Φ_*B*_ would change widely and strongly depend on the initial angle used as the initial condition. After its growing phase when the magnetic moment |**m**| becomes saturated as (*t* − *t*_0_)/τ_0_ > O(1) in Equation (20), the angle Φ_*B*_ would approach a finite, non-zero average value. Evidently, the angle Φ_*B*_ would be small [i.e., the magnetic field **B** would be nearly aligned with the symmetry axis *z*, as clearly indicated by the torque equation (32)], if the magnetic moment |**m**| is sufficiently large. It should be noted that, while we have chosen a magnetic moment growth in our model, the resulting dynamics would be the same as that of an increasing strength of the magnetic field which is realizable in experimental studies.

**Figure 5:**
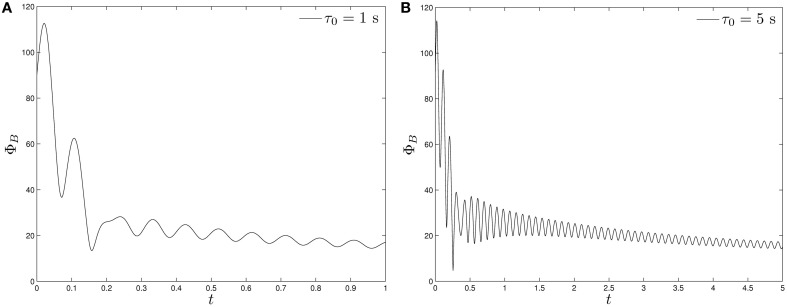
**The angle Φ*_B_* of a swimming rod-shaped magnetotactic bacterium as a function of time starting from Φ*_B_* = 90° under the influence of a rotating magnetic field (Equation 39) with *B*_0_ = 14.00 × 10^−4^ T and Ω_0_ = 2.5133/s: (A) for τ_0_ = 1 s and (B) for τ_0_ = 5 s**.

Starting with the angle Φ_*B*_ = 90° and using the set of the parameters listed in Table 2 together with *B*_0_ = 14.00 × 10^−4^ T and Ω_0_ = 2.5133/s in Equation (39), we perform two computations of solving the twelve coupled equations as an initial-value problem. The first one takes a fast growth rate with τ_0_ = 1 s, the result of which is depicted in Figure 5A. It can be seen that, after changing rapidly and widely during the initial growing phase, the angle Φ_*B*_ approaches the values that are oscillating around 20°. In the second calculation with a slow growth rate with τ_0_ = 5 s, which is shown in Figure 5B, it takes a longer time for the angle Φ_*B*_ to approach an equilibrium value. In general, the external magnetic field **B** is usually not aligned with the magnetic moment **m** and the average equilibrium of the angle Φ*_B_* is primarily determined by the torque equation (32).

**Figure 6:**
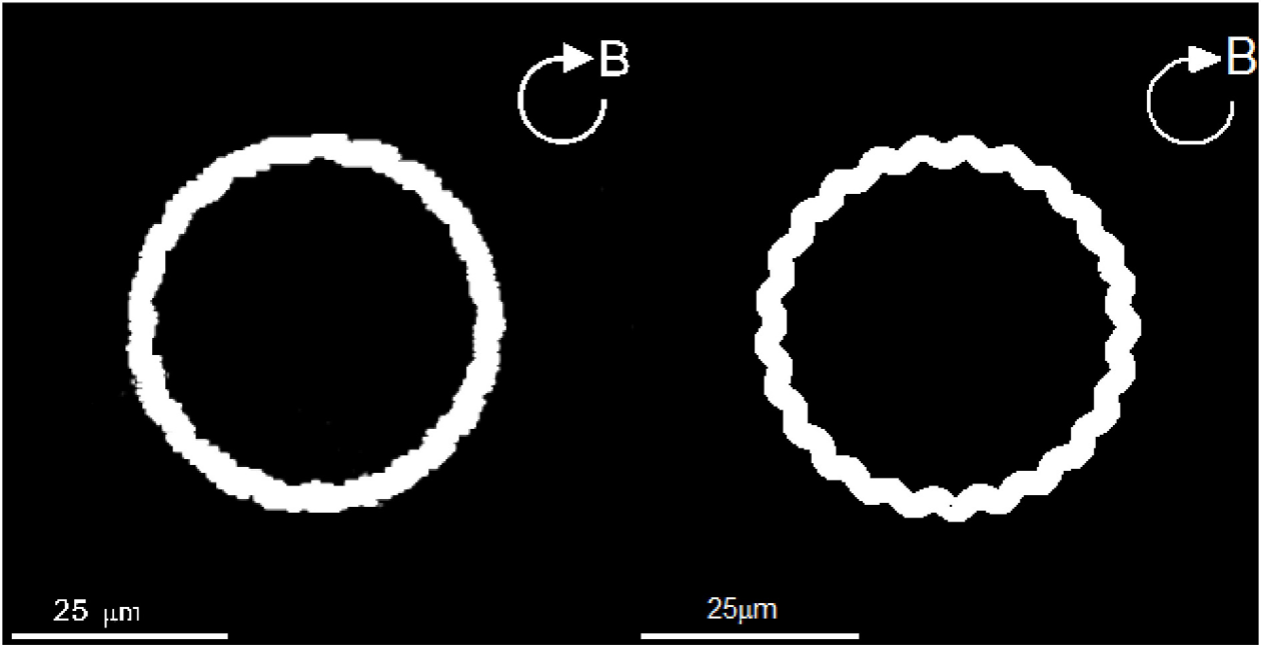
**The swimming trajectories of a rod-shaped magnetotactic bacterium under the influence of an externally imposed rotating magnetic field (Equation 39) with *B*_0_ = 14.00 × 10^−4^ T and Ω_0_ = 2.5133/s**. The **left** panel is recorded in our laboratory experiment with a charge-coupled device camera while the **right** panel shows a numerical solution of the twelve coupled equations using the parameters listed in Table 2.

In contrast to the angle Φ*_B_*, direct comparison can be readily made between the laboratory trajectories of the swimming motion and the corresponding theoretical trajectories. In laboratory experiments, we record the trajectories of a swimming rod-shaped magnetotactic bacterium found in Lake Miyun by using a charge-coupled device camera. Two laboratory experiments and their corresponding theoretical experiments are carried out. In the first laboratory experiment, we impose a rotating magnetic field given by Equation (39) with *B*_0_ = 14.0 × 10^−4^ T with Ω_0_ = 2.5133/s whose trajectories of swimming motion are depicted on the left panel of Figure 6. It is estimated that the average radius of the trajectories of circular path is about 20.0 × 10^−6^ m. Theoretically, we employ the set of the parameters listed in Table 2, together with *B*_0_ = 14.00 × 10^−4^ T and Ω_0_ = 2.5133/s, to solve the twelve coupled equations as a function of time with a moderate value of τ_0_. The right panel of Figure 6 shows the theoretical trajectories of swimming motion for a rod-shaped magnetotactic bacterium after (*t* − *t*_0_)/τ_0_ » 1, which give rise to the average radius of the circular path about 20.5 × 10^−6^ m. It is demonstrated that, for rod-shaped magnetotactic bacteria, the laboratory observations can be largely reproduced by the solutions of our theoretical model using a set of appropriate parameters.

**Figure 7:**
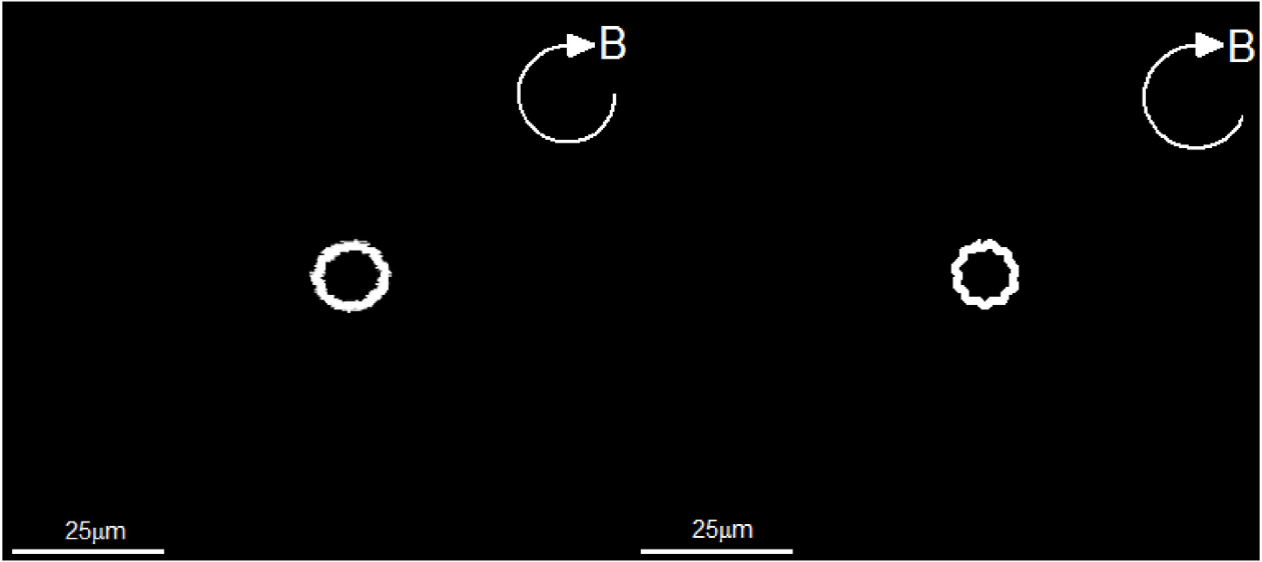
**The swimming trajectories of a rod-shaped magnetotactic bacterium under the influence of a rotating magnetic field (Equation 39) with *B*_0_ = 4.00 × 10^−4^ T and Ω_0_ = 6.28/s**. The **left** panel is recorded in our laboratory experiment with a charge-coupled device camera while the **right** panel shows a numerical solution of the twelve coupled equations using the parameters listed in Table 2.

Since powers of the flagellar motor for a rod-shaped magnetotactic bacterium is fixed, we anticipate that the average radius of circular path would decrease when the rotating frequency Ω_0_ of the externally imposed magnetic field increases. In the second experiment, we also impose a rotating magnetic field given by Equation (39) but with *B*_0_ = 4.0 × 10^−4^ T and an increased frequency Ω_0_ = 6.28/s. The resulting trajectories of swimming motion recorded in our laboratory experiment are depicted on the left panel of Figure 7. It is estimated, from the laboratory observations, that the average radius of of circular path decreases to about 5.2 × 10^−6^ m. The expected decrease is successfully reproduced by the theoretical trajectories of swimming motion, which is depicted on on the right panel of Figure 7. Employing the same set of the parameters listed in Table 2 together with *B*_0_ = 4.00 × 10^−4^ T and Ω_0_ = 6.28/s, the theoretical estimate of the average radius from our computation is about 5.1 × 10^−6^ m.

## 5. Summary and remarks

Through both theoretical and experimental methods, we have investigated the swimming motion of rod-shaped magnetotactic bacteria in a viscous liquid under the influence of an externally imposed, rotating magnetic field. It is shown that a fully three-dimensional Stokes flow, driven by the translation and rotation of a swimming rod-shaped bacterium, exerts the complicated viscous drag and torque on the swimming motion. Under the major assumptions that (1) the body of the bacterium is non-deformable, (2) inertial effects are negligible, and (3) interactions between different bacteria are weak and negligible, we have derived a new system of the twelve coupled equations governing both the motion and orientation of a swimming rod-shape magnetotactic bacterium. Of the twelve coupled equations, (25–27) provide the velocity **v**_*L*_ in the laboratory frame, (Equations 28–30) are for the position of the bacterium in the laboratory frame, (Equations 33–35) give its rotation vector **Ω** and (Equations 36–38) describe the orientation of the bacterium in the laboratory frame.

Using rod-shaped magnetotactic bacteria collected from Lake Miyun near Beijing, China, we have demonstrated that the theoretical swimming patterns described by solutions of the twelve coupled equations are largely similar to those observed in our laboratory experiments under the influence of externally imposed rotating magnetic fields. Despite a good agreement achieved between the theory and the experiments, the weakest component in our theoretical model is perhaps the assumption of non-deformable character of the rod-shaped bacteria under strong viscous and magnetic torques. However, modeling the swimming motion of deformable rod-shaped magnetotactic bacteria without fully understanding how/why rod-shaped magnetotactic bacteria are deformable is, both mathematically and biologically, highly challenging.

## Conflict of interest statement

The authors declare that the research was conducted in the absence of any commercial or financial relationships that could be construed as a potential conflict of interest.
